# Supplementary material for: Tough soldering for stretchable electronics by small-molecule modulated interfacial assemblies
Source: Nat Commun. 2023 Nov 24;14:7723. doi: 10.1038/s41467-023-43574-8 (PMC10673831; doi:10.1038/s41467-023-43574-8)
Supplement: Supplementary file 1 — Supplementary Information [file 41467_2023_43574_MOESM1_ESM.pdf]

Supplementary Information

Tough soldering for stretchable electronics by small-molecule modulated interfacial assemblies

Liqing Ai[1,5], Weikang Lin[2,3,5], Chunyan Cao[1,5], Pengyu Li[2,3], Xuejiao Wang[1], Dong Lv[1], Xin Li[1], Zhengbao Yang[*2,3], and Xi Yao[*1,4]

[1] Department of Biomedical Sciences

City University of Hong Kong

Hong Kong, 999077, China.

[2] Department of Mechanical & Aerospace Engineering

Hong Kong University of Science and Technology

Hong Kong, 999077, China

[3] Department of Mechanical Engineering

City University of Hong Kong

Hong Kong, 999077, China.

[4] City University of Hong Kong Shenzhen Research Institute

Shenzhen, 518000, China.

[5] These authors contributed equally: Liqing Ai, Weikang Lin, Chunyan Cao.

[*] E-mail: zb.yang@cityu.edu.hk; xi.yao@cityu.edu.hk

Supplementary Note

Synthesis of 2-(6-isocyanato-hexylamino)-6-methyl-4[1H]-pyrimidinone (UPy-NCO): UPy-NCO was synthesized according to the literature[1]. 2-Amino-4-hydroxy-6-methylpyrimidine (10 g, 79.9 mmol) was added to a 250 mL round bottomed flask. Hexamethylene diisocyanate (HMDI, 100 mL, 624 mmol) and pyridine (7 mL) were then added, the flask fitted with a reflux condenser, and the mixture stirred at 100˚C overnight under dry nitrogen. Pentane (30 mL) was then added and the solid product, a white powder, was collected by filtration. The solid product was washed 3 times with 125 mL portions of acetone to remove unreacted HMDI and then dried overnight under high vacuum at 60˚C (yield 95%). ^{1}H-NMR (400 MHz; $CDCl_3$): δ = 13.14 (s, 1H, CH_3-C-N$\underline{H}$), 11.87 [s, 1H, CH_2-NH-(C=O)-N$\underline{H}$], 10.19 [t, 1H, CH_2-N$\underline{H}$-(C=O)-NH], 5.82 (s, 1H, C$\underline{H}$=C-CH_3), 3.05-2.98 [m, 4H, NH-(C=O)-NH-C$\underline{H}_2$, C$\underline{H}_2$-NCO], 2.21 (s, 3H, C$\underline{H}_3$), 1.79-1.73 (m, 4H, N-CH_2-C$\underline{H}_2$), 1.07-0.96 (m, 4H, CH_2-CH_2-C$\underline{H}_2$-C$\underline{H}_2$-CH_2-CH_2-NCO) ppm.

Synthesis of UPy-MW$_{PDMSn}$-NH$_2$: UPy-MW$_{PDMSn}$-NH$_2$ with 2000 MWs of poly(dimethylsiloxane) (PDMS) was obtained by reaction of 1 equivalent of UPy-NCO and 10 equivalent of bis(3-aminopropyl) terminated PDMS (MW$_{PDMSn}$-2NH$_2$). Typically, a 500 mL round bottom flask equipped with a reflux cooler was charged with MW$_{PDMS}$2000-2NH$_2$ (20.0 g, 10 mmol) and chloroform (200 mL), then UPy-NCO (0.292 g, 1 mmol) in 20 mL chloroform solution was added dropwise. After reacting at 60˚C under the protection of nitrogen for 6 h, the solvent chloroform was removed, and the solid product was washed 3 times with 125 mL portions of acetone to remove unreacted MW$_{PDMS}$2000-2NH$_2$. UPy-MW$_{PDMS}$2000-NH$_2$ was then collected by filtration and dried overnight under high vacuum at 60˚C (yield 87%). ^{1}H-NMR (400 MHz; $CDCl_3$): δ = 13.14 (s, ^{1}H, CH_3-C-N$\underline{H}$), 11.87 [s, 1H, CH_2-NH-(C=O)-N$\underline{H}$-C], 10.19 [t, 1H, CH_2-N$\underline{H}$-(C=O)-NH-C], 5.37 (s, 1H, C$\underline{H}$=C-CH_3), 3.77 [q, 8H, NH-(C=O)-NH-C$\underline{H}_2$, C$\underline{H}_2$-NH-(C=O)-NH-C$\underline{H}_2$, NH_2-C$\underline{H}_2$-CH_2-CH_2-Si], 2.19 (s, 3H, Ar-C$\underline{H}_3$), 1.62-1.58 (m, 8H, NH-CH_2-C$\underline{H}_2$-CH_2-CH_2-C$\underline{H}_2$-CH_2-NH, NH-CH_2-C$\underline{H}_2$-CH_2-Si, NH_2-CH_2-C$\underline{H}_2$-CH_2-Si), 1.35-1.28 (m, 4H, NH-CH_2-CH_2-C$\underline{H}_2$-C$\underline{H}_2$-CH_2-CH_2-NH), 0.9-0.85 (m, 4H, C$\underline{H}_2$-Si), 0.09-0.05 (m, 48H, C$\underline{H}_3$-Si) ppm.

Other UPy-MW$_{PDMSn}$-NH$_2$ were prepared through similar procedures with corresponding MWs of bis(3-aminopropyl) terminated PDMS.

Stud pull test: As shown in the schematic figure, stud pull test was used as a process control method to determine the adhesion of components and ULPC-TPU substrates. This was achieved by gluing a stud to the top surface of the component and applying perpendicular forces away from the component interface. The mechanical test machine (50 N load cell; Instron-5566) with a constant speed of 100 mm/min. The stud-pull strength was measured according to the maximum perpendicular forces and the contact area of the component and ULPC-TPU.

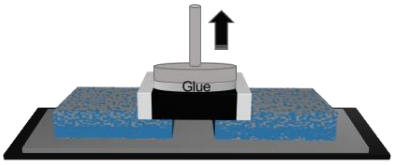

Stud pull test

90° peel-off test: The ULPC samples with the size of 10 mm width and 100 mm length were attached to the Cu film and heated to 110 °C for 5 min. After cooling to room temperature, the separated top component was clamped to grips. The mechanical test machine (50 N load cell; Instron-5566) with a constant speed of 50 mm/min. The peel-off strength was measured according to the forces and the contact width of the Cu films and ULPC.

Shear test: The ULPC samples with the size of 10 mm width and 10 mm length were attached between two Cu films and heated to 110°C for 5 min. After cooling to room temperature, the two Cu films were clamped to grips. The mechanical test machine (50 N load cell; Instron-5566) with a constant speed of 100 mm/min. The shear strength was measured according to the forces and the contact area of the Cu films and ULPC.

General characterizations: Solution ^{1}H NMR spectra were recorded on a Bruker DRX 300 NMR spectrometer in $CDCl_3$ at room temperature (20˚C), and chemical shifts are reported in ppm relative to tetramethylsilane as an internal standard. The microstructures and thickness of the conductive composite were investigated by using a field emission scanning electron microscope (SEM, Philips XL30CP) at 10.0 kV. TEM (Philips Tecnai 12) measurements were performed at a voltage of 100 kV. An energy-dispersive X-ray spectrometer (EDS, Oxford Instruments INCA Energy 200) fitted to SEM was used for elemental analysis. Fourier transform infrared (FT-IR) spectra were tested by an FT-IR spectrometer (Perkin Elmer Spectrum 100), and the samples were prepared by directly casting diluted polymer solution or small-molecule modulated powder onto a NaCl plate to form an ultrathin film. Micro CT Scanner (NIKON XTH 225/320) included data collection and data processing. An X-ray source was used to irradiate the ULPC sample, and a series of radiographs was synthesized by

computer software to reconstruct a three-dimensional (3D) image. The maximum resolution (depending on object size) is 0.6 μm. The semicrystalline properties of the composites were examined under X-ray diffraction (XRD) analysis instrument (Brucker AXS, D2 PHASER) and Small Angle X-ray Diffraction (SAXD) analysis instrument (SAXSess mc2). The Raman measurements were acquired with an alpha 300R WITec confocal Raman system (WITec GmbH, Ulm, Germany). To avoid sample damage during data acquisition, the 532 nm excitation of a frequency-doubled neodymium-doped yttrium-aluminum-garnet (Nd:YAG) laser was kept at a low power output of about 10 mW. The Raman mapping images were acquired with 10 μm*10 μm scan sizes. Optical surface profiler images were detected on a Wyko NT9300 3D surface profiler.

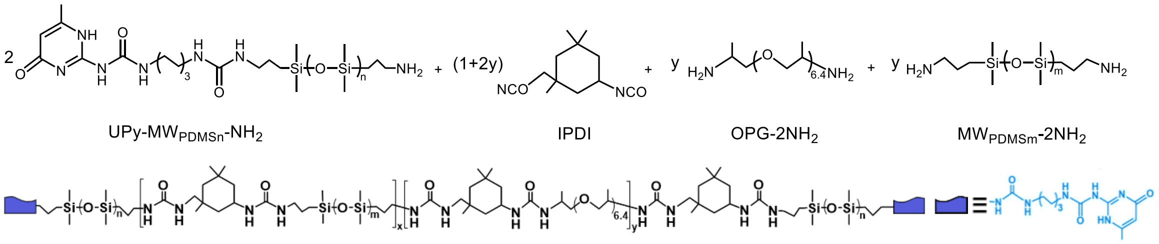

Supplementary Fig. 1 Synthetic route for linear supramolecular polymers through condensation reactions between amino terminated monomers (OPG-2NH$_2$ and MW$_{PDMSm}$-2NH$_2$), UPy precursors (UPy-MW$_{PDMSn}$-NH$_2$), and diisocyanate linkers (IPDI).

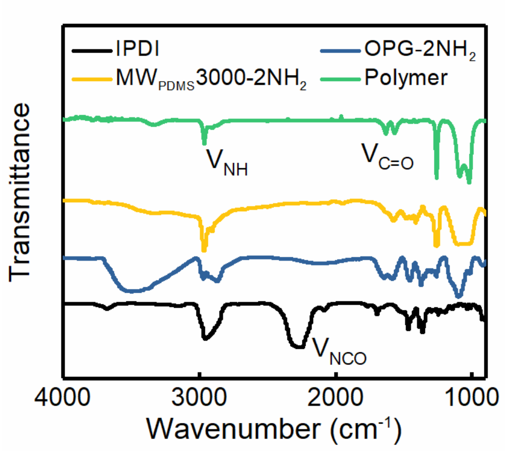

Supplementary Fig. 2 FT-IR spectrum of IPDI, OPG-2NH$_2$, MW$_{PDMS}$3000-2NH$_2$, and polymer (MW$_{PDMS}$2000, UPy0.08). The FT-IR absorption peaks appearing at 1631 and 3420 cm^{-1} represent the stretching vibration of C=O and N-H respectively. The disappearance of the absorption peak of NCO (2250 cm^{-1}) illustrates the complete consumption of reactants.

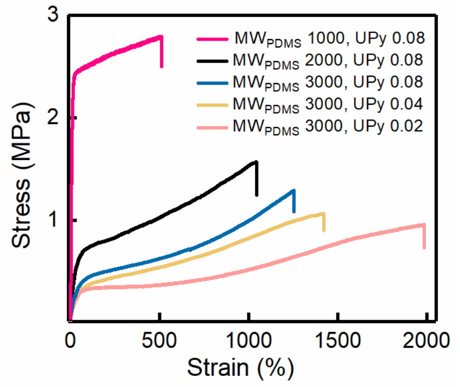

Supplementary Fig. 3 Typical stress-strain curves of different polymers with different MWs of UPy-terminated siloxane and different feeding amounts of UPy monomers.

Supplementary Table 1 Mechanical properties of all polymers.

Sample	Ultimate tensile strength (MPa)	Elongation at break (%)	Young's modulus* (MPa)	Toughness (MJ m^{-3})
MW_{PDMS}1000, UPy0.08	2.8	392	34.0	10.2
MW_{PDMS}2000, UPy0.08	1.6	1045	2.8	11.1
MW_{PDMS}3000, UPy0.08	1.3	1251	1.2	9.3
MW_{PDMS}3000, UPy0.04	1.1	1422	0.8	9.5
MW_{PDMS}3000, UPy0.02	1.0	1986	0.4	11.3

*Young's modulus was calculated by finding the slope of the linear region of the stress-strain graph.

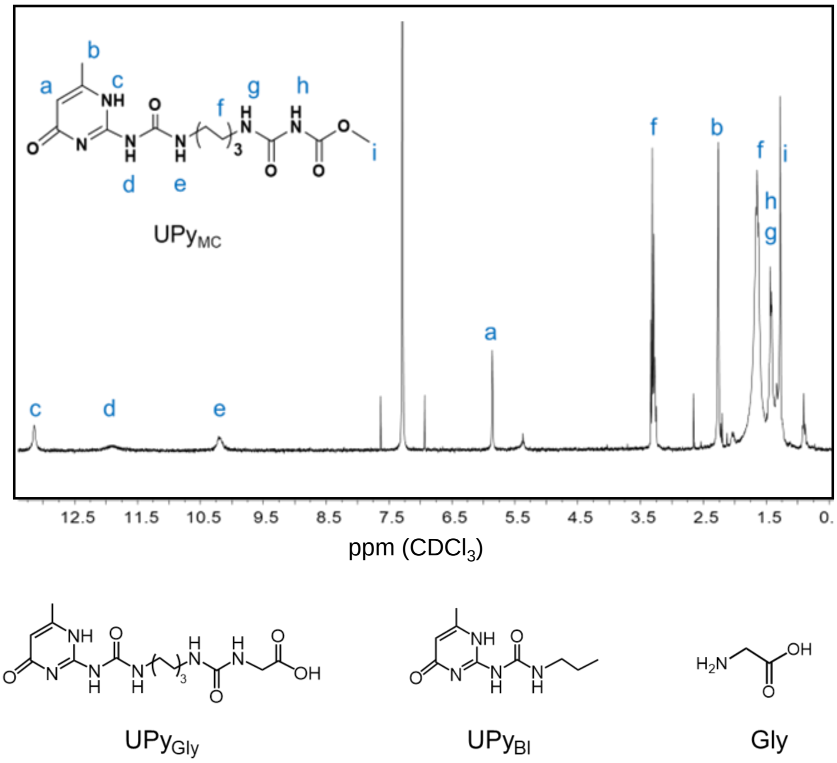

Supplementary Fig. 4 ^{1}H NMR spectrum of UPy$_{MC}$ (MC is the abbreviation for "Methyl Carbamate") and structures of UPy$_{Gly}$ (Gly is the abbreviation for "Glycine"), UPy$_{BI}$ (BI is the abbreviation for "Butyl Isocyanate"), and glycine (Gly) molecules.

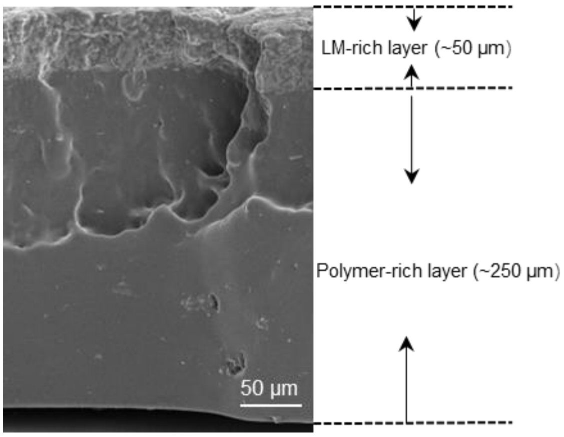

Supplementary Fig. 5 Cross-section SEM image of the ULPC.

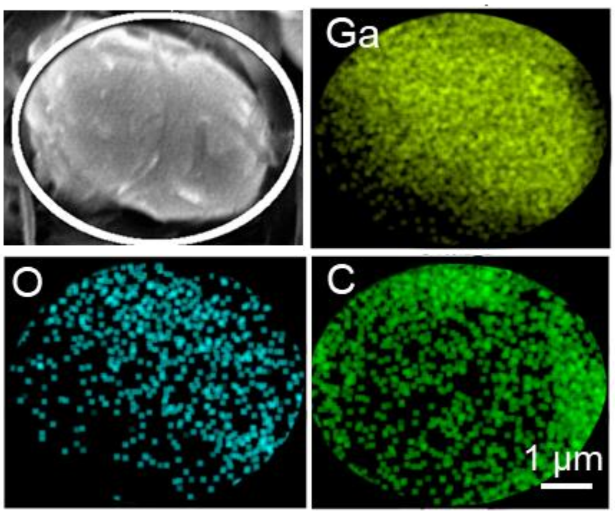

Supplementary Fig. 6 SEM images and energy-dispersive X-ray spectroscopy (EDS) of UPy_{MC} adsorbed on the surface of LMP and its corresponding element mapping (Ga, O, and C), sonication time: ~20 min. It can be seen that C elements are uniformly distributed around the surface of LMP, indicating UPy_{MC} is uniformly coated around the LMP surface.

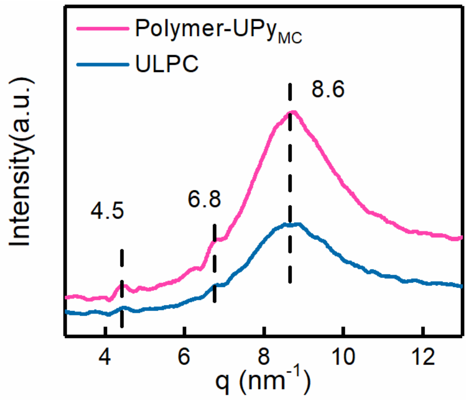

Supplementary Fig. 7 WAXS spectra of polymer-UPy_{MC} (MW_{PDMS}2000, UPy0.08) and ULPC samples.

Supplementary Table 2 Mechanical properties of the developed pristine polymer, polymer-LM, polymer-UPy_{MC}, and as-prepared ULPC with different amounts/types of modulators.

Sample	Ultimate tensile strength (MPa)	Elongation at break (%)	Young’s modulus* (MPa)	Toughness (MJ m^{-3})
Polymer	1.6	1045	2.8	11.1
Polymer-LM	0.7	1062	0.5	5.3
Polymer-2wt%UPy_{MC}	3.0	483	12.8	11.5
ULPC-1wt%UPy_{MC}	2.3	1005	8.8	15.3
ULPC-2wt%UPy_{MC}	3.2	965	10.3	19.8
ULPC-4wt%UPy_{MC}	3.5	843	11.5	18.9
ULPC-6wt%UPy_{MC}	3.1	573	11.6	12.3
ULPC-2wt%UPy_{Gly}	3.4	913	7.2	19.4
ULPC-2wt%UPy_{BI}	1.5	919	2.1	9.1
ULPC-2wt%Gly	0.7	996	1.5	5.3

* Young's modulus was calculated by finding the slope of the linear region of the stress-strain graph.

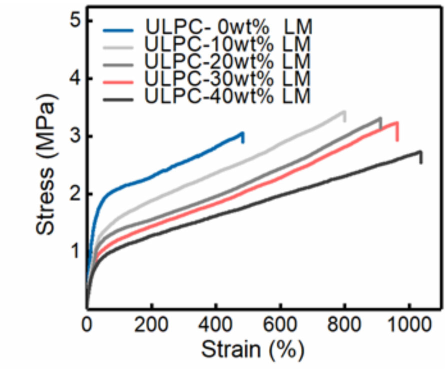

Supplementary Fig. 8 Comparison of the tensile stress-strain curves for the composites prepared by different LM content. Polymers were prepared from siloxane oligomers with ~2000-MW and 0.08 amount of UPy monomers, and ULPC was fabricated from the polymer with specific LM content and 2 wt% UPy_{MC}.

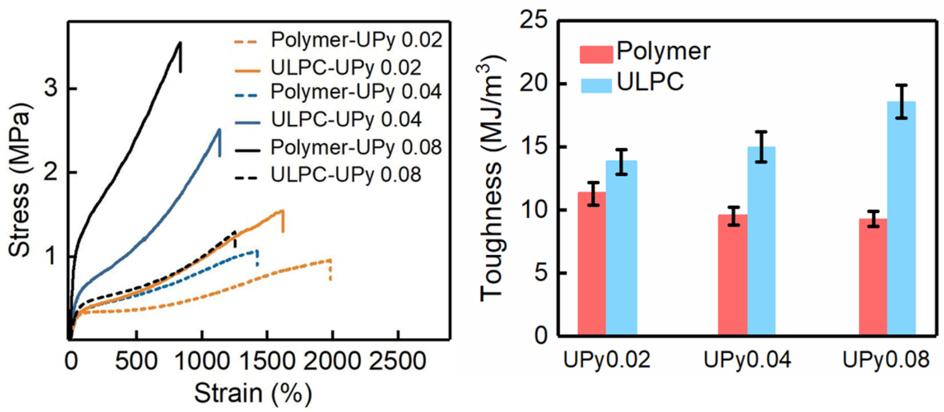

Supplementary Fig. 9 Typical stress-strain curves of polymers with different contents of UPy monomers and their ULPC samples (left), and the comparison of their toughness (right).

Supplementary Table 3 Mechanical properties of polymers with different contents of UPy monomers and their ULPC samples.

Sample	Ultimate tensile strength (MPa)	Elongation at break (%)	Young's modulus* （MPa)	Toughness (MJ m^{-3})
Polymer-UPy0.02	1.0	1986	0.4	11.3
ULPC-UPy0.02	1.54	1618	1.6	13.8
Polymer-UPy0.04	1.1	1422	0.8	9.5
ULPC-UPy0.04	2.5	1136	2.6	15.0
Polymer-UPy0.08	1.3	1251	1.2	9.3
ULPC-UPy0.08	3.5	834	7.7	18.5

* Young's modulus was calculated by finding the slope of the linear region of the stress-strain graph.

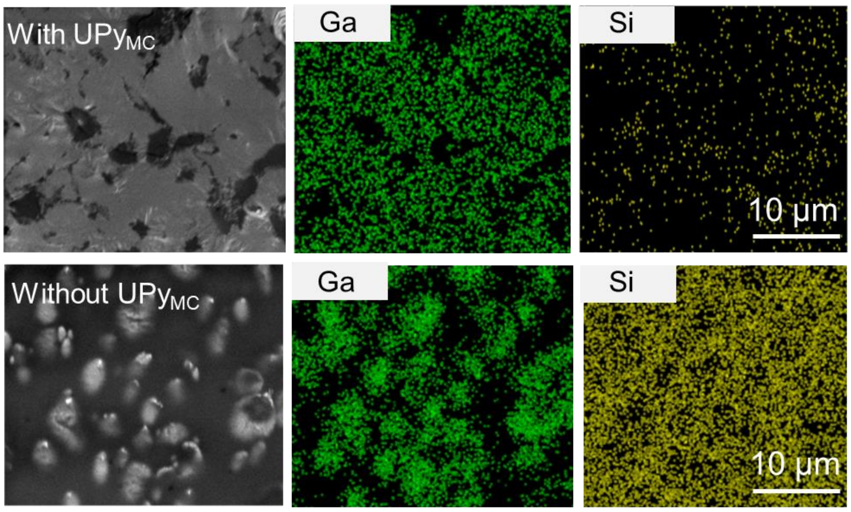

Supplementary Fig. 10 SEM images and the corresponding element mapping (Ga, Si) of the LM-rich surface of composites with or without UPy$_{MC}$ modulators. LMPs show a continuous form with UPy$_{MC}$, in comparison, in the case without UPy$_{MC}$, there is limited coalescence during evaporation, and the LMPs maintain the sphere-like morphology without connections in LM-rich surface.

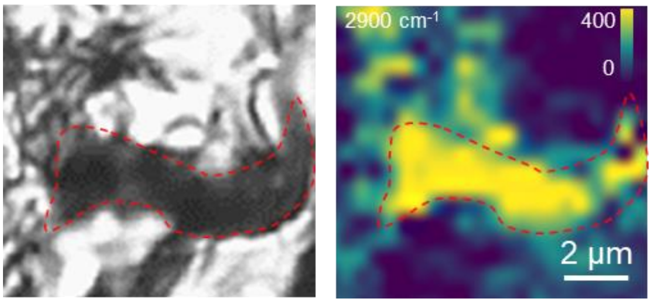

Supplementary Fig. 11 Optical images of LM-rich surface of ULPC and its corresponding Raman mapping. The distribution of the 2900 cm^{-1} peaks, which are assigned to the asymmetric and symmetric stretches of the C-H in PDMS backbone[2], indicates the distribution of the polymer-rich domain.

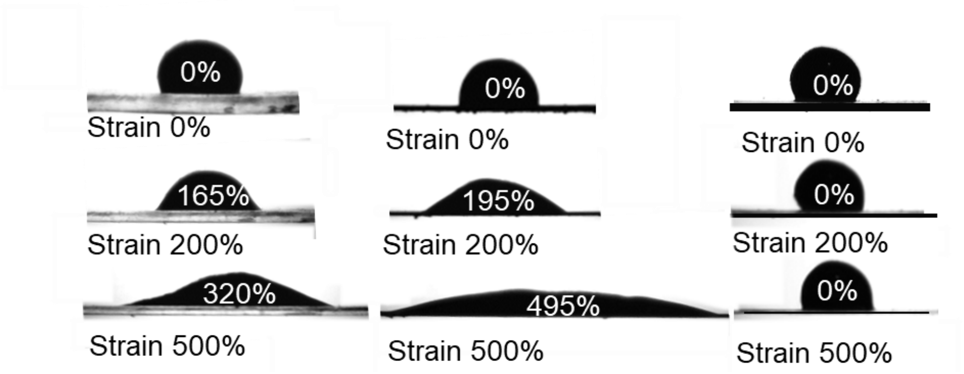

Polymer ULPC Treated with HCl

Supplementary Fig. 12 Snapshots showing the shape evolution of a LM droplet (before and after acid treatment) depositing on the polymer matrix and UPLC under continuous strains from 0% to 500%.

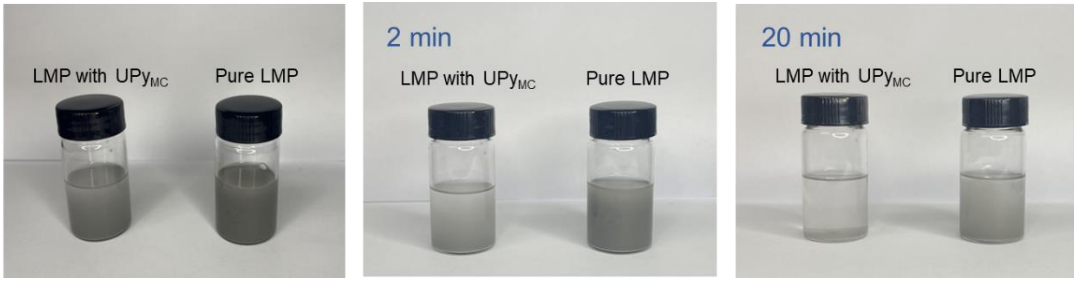

Supplementary Fig. 13 Time-dependent stability of LMP with (right) or without (left) UPy_{MC} in dichloromethane (DCM) solution for 20 min. LMP with UPy_{MC} shows obvious precipitation within 2 min. While the pure LMP dispersion is still cloudy for 20 min (sonication time: 5 min). In the absence of UPy_{MC} modulators, the LMP takes longer to settle to the bottom during the solvent evaporation process. This indicates that a smaller amount of LMP is settled at the bottom, resulting in a less compact assembly. As a result, after peeling off, the LMP particles cannot form an interconnected network, hindering the formation of a conductive path.

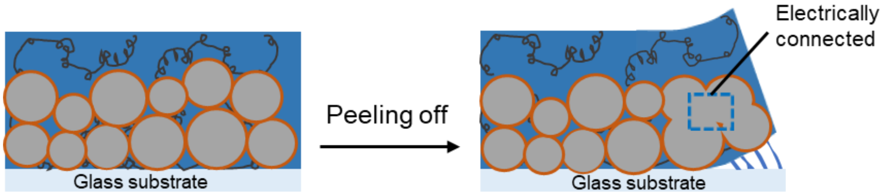

Supplementary Fig. 14 Schematic illustration of the peeling-induced activation process. During the peeling process, a sufficient amount of stress is applied to the particles, allowing for the percolation of electrical activation.

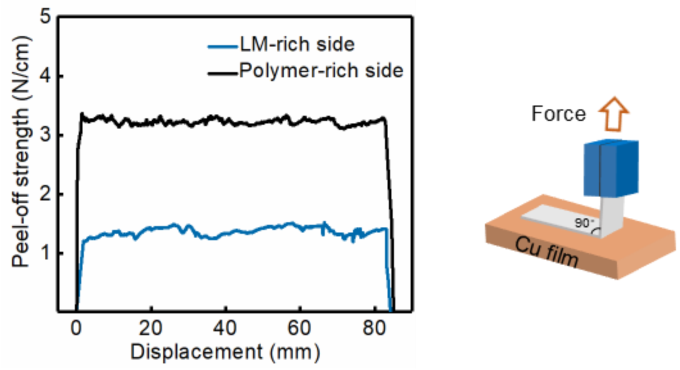

Supplementary Fig. 15 The tensile curves for the 90° peel-off test. The pull-off strength was measured according to the forces and the contact area of the Cu films and ULPC with 30 wt% LM content.

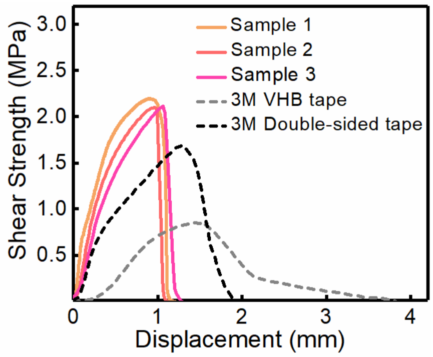

Supplementary Fig. 16 Representative tensile curves for shear strength test. The shear strength was measured according to the forces and the contact area of the Cu films and samples. Samples 1-3 are duplicated ULPC samples with 30 wt% LM content. 3M VHB tape 5952 and 3M double-sided tape 9448A are used as control. From the result, the maximum shear strength of the ULPC sample was 2.1±0.08 MPa, which shows competitive adhesion performance with double-sided tape and was superior to VHB tape.

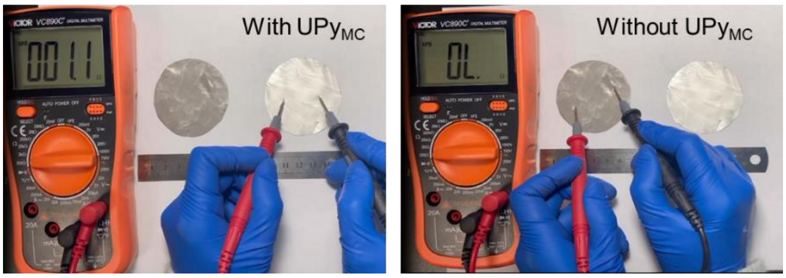

Supplementary Fig. 17 The resistance measurement of the LM-rich surface of the composites with or without adding UPy$_{MC}$ modulators.

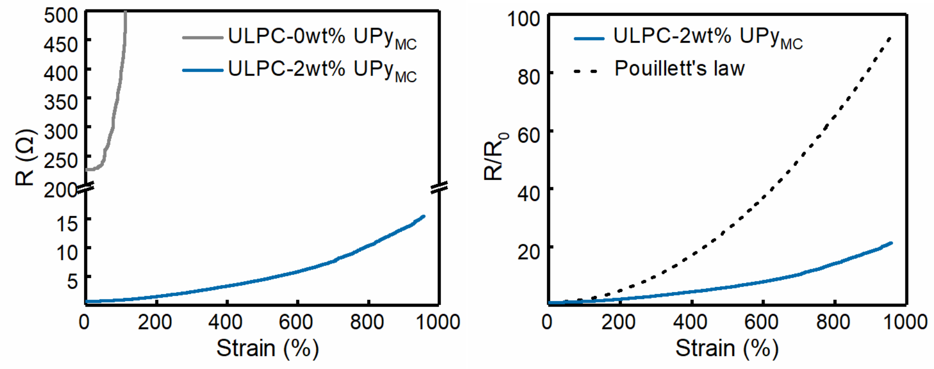

Supplementary Fig. 18 Comparison of relative resistance change over strains with or without adding UPy$_{MC}$ modulators (left). Normalized change in resistance as a function of strain (gradient of blue solid lines) along with the theoretical prediction using Pouillet's law for an incompressible elastomer with constant volumetric resistivity (dashed black line) (right). The resistance changes at small strains (0~100%) agree well with Pouillet's law.

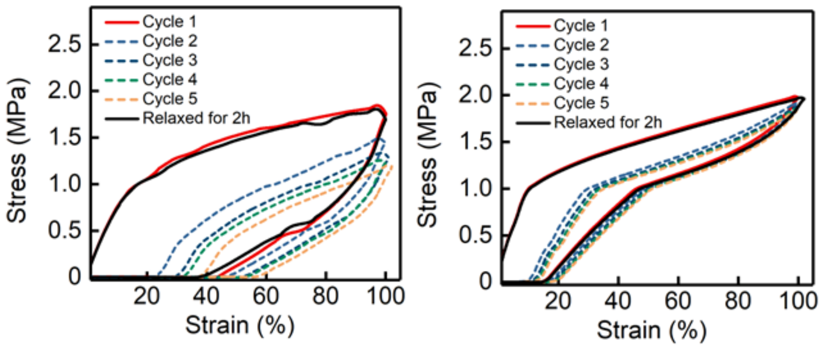

Supplementary Fig. 19 Fatigue resistance of ULPC (left) and ULPC-TPU film (right) cyclic stress-strain curves over five times successive loading/unloading processes without rest, and the 6th cycle after resting at room temperature for 2h. The hysteresis loop area in the second cycle is significantly reduced because the ruptured sacrificial bonds in the first cycle did not have enough time to be reconstructed to their original state. This downward trend slightly decreased in the sequential cycles, implying continuous reorganization of the sacrificial bonds. After being rested at room temperature for 2h, the sample shows a similar loading/unloading curve to the original one, indicating the good fatigue resistance of ULPC-TPU film.

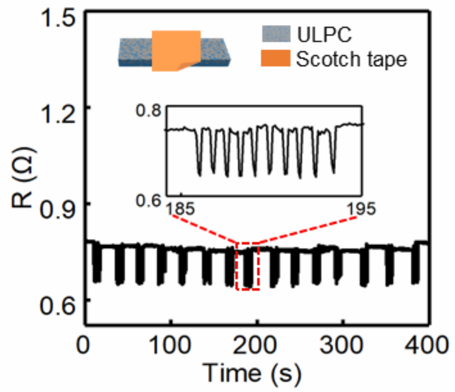

Supplementary Fig. 20 Resistance changes of the LM-rich layer under 150 cycles of peeling using Scotch tapes. The inset shows a group of ten continuous stick-peeling tests.

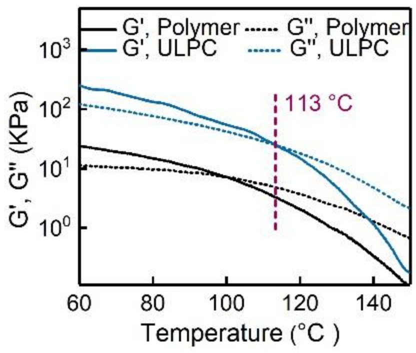

Supplementary Fig. 21 Rheological testing of polymer and ULPC samples. At low temperatures, G′ is larger than G″, showing that the elastic property of ULPC is dominant. Upon increasing temperatures, G′ decreases faster than G″, and the viscous property of ULPC is predominant at the region of temperature > 113°C.

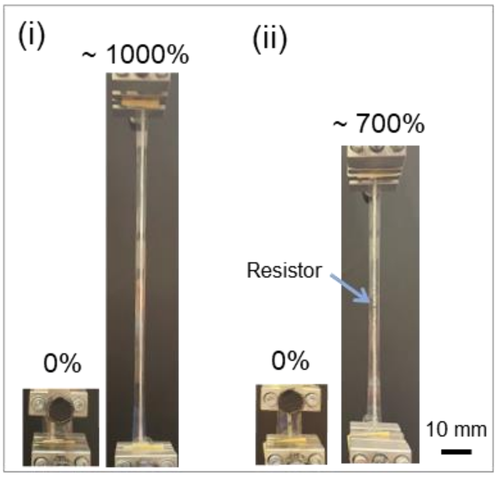

Supplementary Fig. 22 Comparison of the maximum stretching of the i) bare ULPC-TPU and ii) the ULPC-TPU welded with a zero-ohm resistor.

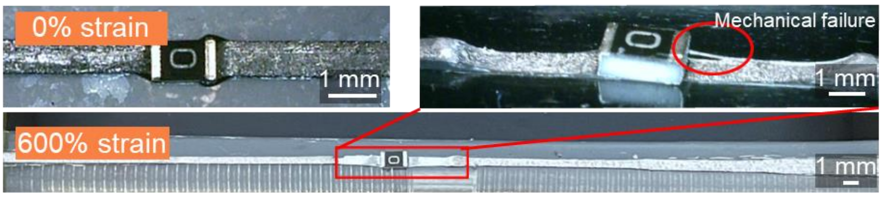

Supplementary Fig. 23 Uniaxial tensile strain of the ULPC-TPU with an embedded zero-ohm resistor to 600%.

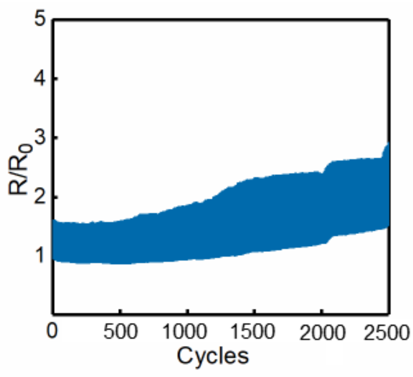

Supplementary Fig. 24 Resistance stability of chip-integrated ULPC-TPU in 2500 stretch-release cycles at 100% strain.

Supplementary Table 4 Conductivity, stretchability (with or without the chip), change of resistance (R/R_0), adhesion, encapsulation of various LM-based stretchable composites.

Ref.	Composites	Stretchability (%)	Stretchability with chip (%)	R/R_0	Adhesion (N/cm)	Encapsulation
**Our**	**ULPC**	**965**	**700**	**20**	**1.6***	**No need**
[3][3]	SIS-LM	1200	700	50	<0.1	Need
[4][4]	bGaIn	1200	500	2	/	No need
[5][5]	SBS-LM	500	200	500	0.3_{MPa}	No need
[6][6]	PSA-LM	950	200	10	1.8	No need
[7][7]	Fe-LM	300	200	18	/	Need
[8][8]	Sticker-LM	/	100	2	0.5_{MPa}	No need
[9][9]	SWCNT-LM	/	30	1.1	/	No need
[10][10]	LM SBS mat	2200	/	1	/	/
[11][11]	Cu-LM	1200	/	40	/	/
[12][12]	TPU-LM	1000	/	20	<0.1	/
[13][13]	HRHP-LM	950	/	20	0.35	/
[14][14]	SEBS-LM	800	/	30	/	/
[15][15]	PVDF-LM	700	/	400	/	/
[16][16]	PDMS-LM	500	/	2	/	/
[17][17]	PU-LM	500	/	1	/	/
[18][18]	Ni-LM	350	/	7	/	/
[19][19]	CNF-LM	250	/	100	/	/
[20][20]	STICK-LM	100	/	1	0.9	/
[21][21]	PVA-LM	100	/	8	/	/
[22][22]	TA-LM	70	/	2	/	/

* The adhesion was measured by the 90° pull-off test of LM-rich side of ULPC with 30 wt% LM content.

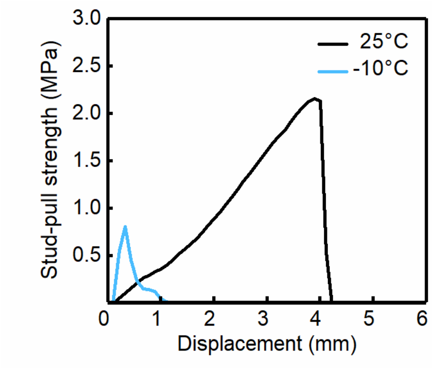

Supplementary Fig. 25 Comparison of the stud-pull strength of ULPC-TPU at 25°C (room temperature) and cooled to -10°C for 20 min.

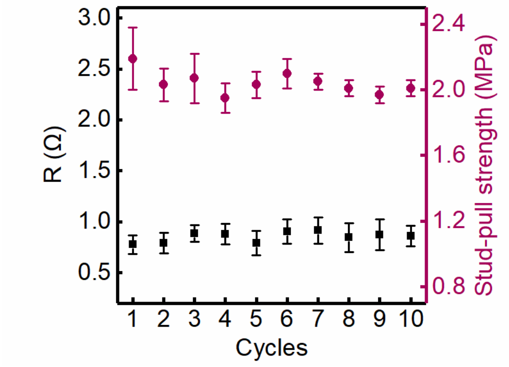

Supplementary Fig. 26 The changes of resistance and maximum stud-pull strength of zero-ohm resistors in ULPC-TPU over repeated substitution cycles.

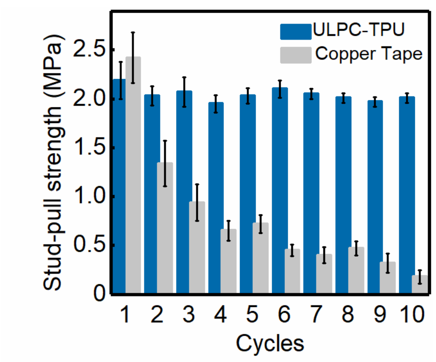

Supplementary Fig. 27 Comparison of maximum stud-pull strength over repeated peeling-substitution cycles of ULPC-TPU with commercial conductive copper tape.

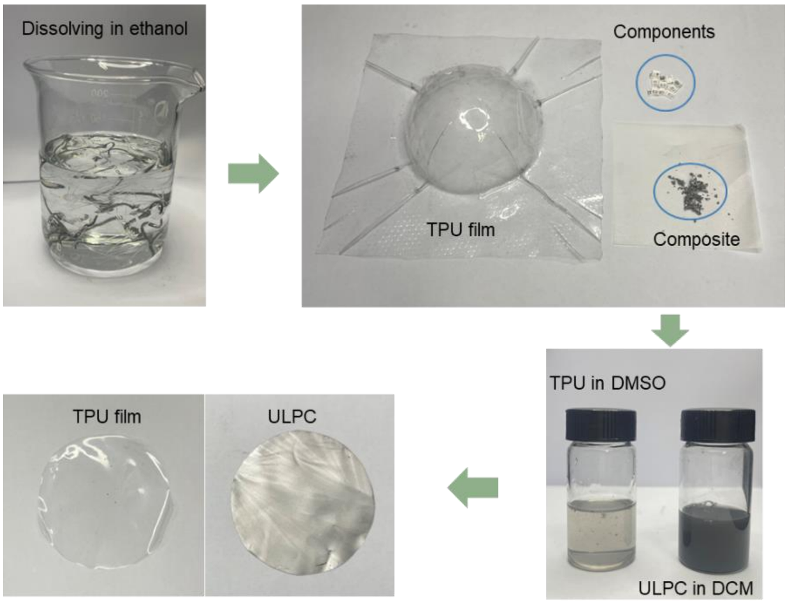

Supplementary Fig. 28 Recycling process of an integrated 3D circuit. The circuit is placed into an ethanol solution, the ULPC could be easily dissolved and the TPU didn't dissolve. In this way, the ink and the components are separated from the TPU substrate. Alternatively, after removing the components, the concentrated ink can be dissolved in dichloromethane (DCM) and placed to sonicate for 1 min to ensure uniform dispersion and the 3D TPU film can be dissolved in dimethylsulfoxide (DMSO). Then poured into glass molds to re-obtain ULPC and TPU film separately.

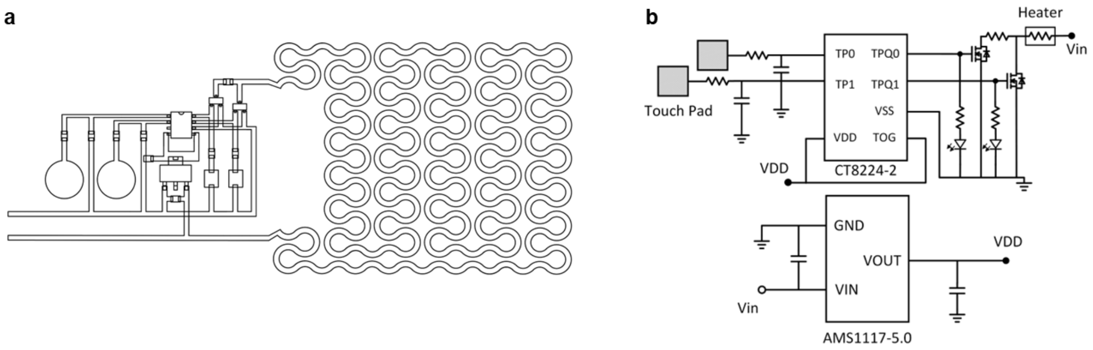

Supplementary Fig. 29 a, The design of the 3D circuit, **b,** diagram of the summing amplifier circuit.

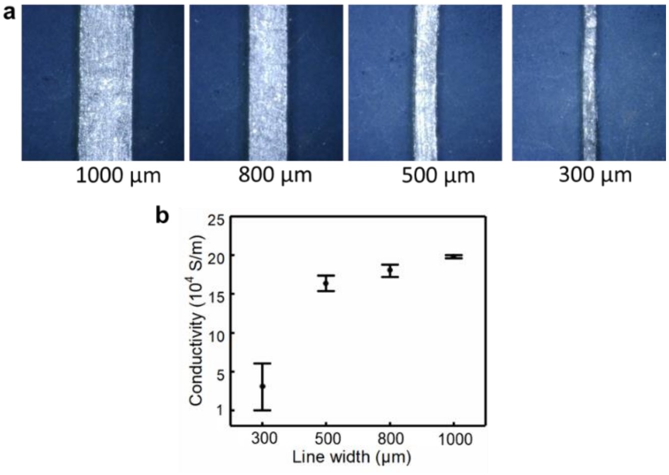

Supplementary Fig. 30 ULPC with different line widths. **a**, Optical image of ULPC lines with different line widths. **b**, Conductivity of ULPC line with different line widths.

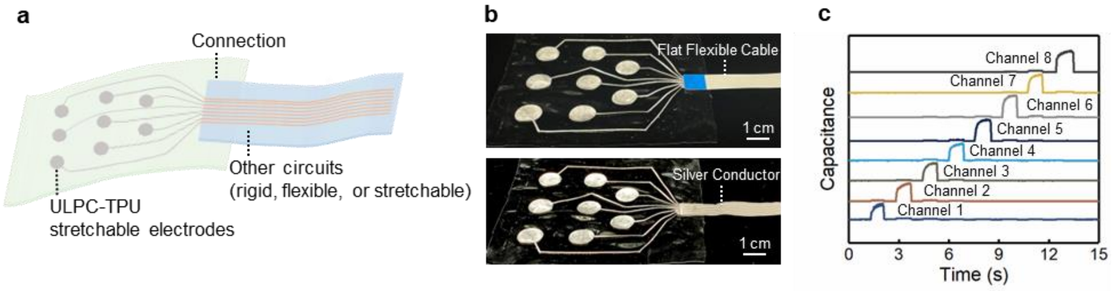

Supplementary Fig. 31 An 8-channel capacitive sensor array assembled by ULPC solder connection. **a**, Schematic of the application of our solder with other circuits (rigid, flexible, or stretchable) as a stretchable connector. **b**, Photograph of a ULPC-TPU 8-channel capacitive sensor array, consisting of three parts: stretchable electrodes, flat flexible cables/ flexible silver conductors (700 μm linewidth and 1 mm pitch), and the printed circuit board. **c**, Capacitive signal from 8 channels obtained from ULPC electrodes when we touch the capacitive touch pad of each channel in turn. The stable and low-impedance connection interface ensures accurate output signals reflecting the capacitive change of each channel with low crosstalk and noise.

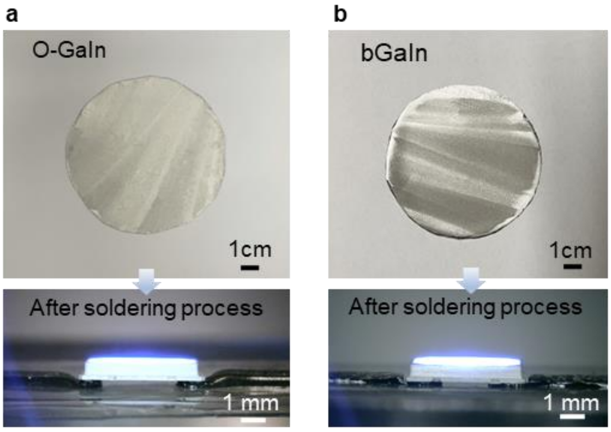

Supplementary Fig. 32 Optical images of prepared **a**, O-GaIn[23] and **b**, bGaIn[4] polymer composites and they integrated with LED chip by the soldering process.

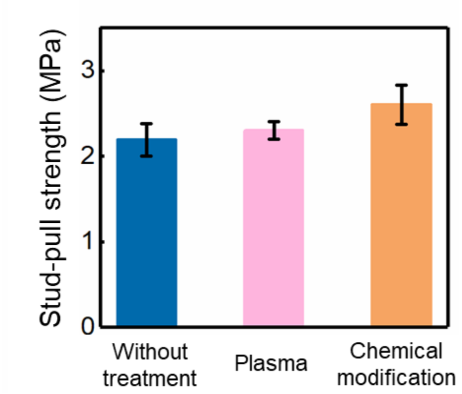

Supplementary Fig. 33 Comparison of the maximum stud-pull strength by different surface treatment approaches. The plasma was conducted with 40 W/ 3 min, the chemical modification of hydroxyl was put components in ethanol ultrasound for 5 min, then placed in a mixture of sodium hydroxide and ethanol (1:1) ultrasound for 5 min, and dried with nitrogen.

Supplementary references

1. Liu, M., Liu, P., Lu, G., Xu, Z. & Yao, X. Multiphase-assembly of siloxane oligomers with improved mechanical strength and water-enhanced healing. *Angew. Chem. Int. Ed. Engl.* **57**, 11242-11246 (2018).

2. Jayes, L., Hard, A.P., Séné, C., Parker, S.F. & Jayasooriya, U.A. Vibrational Spectroscopic Analysis of Silicones: A Fourier Transform-Raman and Inelastic Neutron Scattering Investigation. *Anal. Chem.* **75**, 742-746 (2003).

3. Lopes, P.A., Santos, B.C., de Almeida, A.T. & Tavakoli, M. Reversible polymer-gel transition for ultra-stretchable chip-integrated circuits through self-soldering and self-coating and self-healing. *Nat. Commun.* **12**, 4666 (2021).

4. Liu, S., Shah, D.S. & Kramer-Bottiglio, R. Highly stretchable multilayer electronic circuits using biphasic gallium-indium. *Nat. Mater.* **20**, 851-858 (2021).

5. Mou, L. *et al.* Highly stretchable and biocompatible liquid metal-elastomer conductors for self-healing electronics. *Small* **16**, 2005336 (2020).

6. Tang, L., Yang, S., Zhang, K. & Jiang, X. Skin Electronics from Biocompatible In Situ Welding Enabled By Intrinsically Sticky Conductors. *Adv. Sci.* **9**, e2202043 (2022).

7. Guo, R., Sun, X., Yuan, B., Wang, H. & Liu, J. Magnetic liquid metal (Fe-EGaIn) based multifunctional electronics for remote self-healing materials, degradable electronics, and thermal transfer printing. *Adv. Sci.* **6**, 1901478 (2019).

8. Kim, M., Park, J.J., Cho, C. & Ko, S.H. Liquid Metal based Stretchable Room Temperature Soldering Sticker Patch for Stretchable Electronics Integration. *Adv. Funct. Mater.* 03286 (2023).

9. Oh, E. *et al.* Highly reliable liquid metal-solid metal contacts with a corrugated single-walled carbon nanotube diffusion barrier for stretchable electronics. *Adv. Funct. Mater.* **28**, 1806014 (2018).

10. Ma, Z. *et al.* Permeable superelastic liquid-metal fibre mat enables biocompatible and monolithic stretchable electronics. *Nat. Mater.* **20**, 859-868 (2021).

11. Li, Y. *et al.* Ultrasensitive and ultrastretchable electrically self-healing conductors. *Proc. Natl. Acad. Sci. USA* **120**, e2300953120 (2023).

12. Tang, L., Mou, L., Zhang, W. & Jiang, X. Large-Scale Fabrication of Highly Elastic Conductors on a Broad Range of Surfaces. *ACS Appl. Mater. Interfaces* **11**, 7138-7147 (2019).

13. Yang, L. *et al.* Self-healing, reconfigurable, thermal-switching, transformative electronics for health monitoring. *Adv. Mater.* **35**, 2207742 (2023).

14. Wang, S. *et al.* Intrinsically stretchable electronics with ultrahigh deformability to monitor dynamically moving organs. *Sci. Adv.* **8**, 5511 (2022).

15. Zheng, L. *et al.* Conductance-stable liquid metal sheath-core microfibers for stretchy smart fabrics and self-powered sensing. *Sci. Adv.* **7**, 4041 (2021).

16. Yao, B. *et al.* Highly stretchable polymer composite with strain-enhanced electromagnetic

interference shielding effectiveness. *Adv. Mater.* **32**, 1907499 (2020).

17. Xu, Y. *et al.* Porous liquid metal-elastomer composites with high leakage resistance and antimicrobial property for skin-interfaced bioelectronics. *Sci. Adv.* **9**, 0575 (2023).

18. Daalkhaijav, U., Yirmibesoglu, O.D., Walker, S. & Mengüç, Y. Rheological Modification of Liquid Metal for Additive Manufacturing of Stretchable Electronics. *Adv. Mater. Technol.* **3**, 1700351 (2018).

19. Li, X. *et al.* Evaporation-induced sintering of liquid metal droplets with biological nanofibrils for flexible conductivity and responsive actuation. *Nat. Commun.* **10**, 3514 (2019).

20. Haque, A.B.M.T. *et al.* Electrically Conductive Liquid Metal Composite Adhesives for Reversible Bonding of Soft Electronics. *Adv. Funct. Mater.* (2023).

21. Zhao, Y. *et al.* A self-healing electrically conductive organogel composite. *Nat. Electron.* **6**, 206-215 (2023).

22. Rahim, M.A. *et al.* Polyphenol-Induced Adhesive Liquid Metal Inks for Substrate-Independent Direct Pen Writing. *Adv. Funct. Mater.* **31** (2020).

23. Wang, X. *et al.* Printed Conformable Liquid Metal e-Skin-Enabled Spatiotemporally Controlled Bioelectromagnetics for Wireless Multisite Tumor Therapy. *Adv. Funct. Mater.* **29** (2019).
